# Role of Mast Cells and Neuroinflammation in Neuropsychiatric Disorders of the Developmental Period

**DOI:** 10.3390/biom16040530

**Published:** 2026-04-02

**Authors:** Ernesto Aitella, Ludovico Neri, Gianluca Azzellino, Ciro Romano, Massimo De Martinis, Rita Roncone, Lia Ginaldi

**Affiliations:** 1 Department of Life, Health and Environmental Sciences, University of L’Aquila, 67100 L’Aquila, Italy; ludovico.neri@graduate.univaq.it (L.N.); gianluca.azzellino@graduate.univaq.it (G.A.); rita.roncone@univaq.it (R.R.); 2 Allergy and Clinical Immunology Unit, Center for the Diagnosis and Treatment of Osteoporosis, AUSL 04 Teramo, 64100 Teramo, Italy; 3 Neurology and Psychiatry Unit for Children and Adolescents, ASL 1 Abruzzo Avezzano–Sulmona–L’Aquila, 67100 L’Aquila, Italy; 4 Complex Operational Unit, Adriatic District Area, AUSL 04 Teramo, 64100 Teramo, Italy; 5 Clinical Immunology Outpatient Clinic, Division of Internal Medicine, Department of Advanced Medical and Surgical Sciences, “Luigi Vanvitelli” University of Campania, 80131 Naples, Italy; ciro.romano@unicampania.it; 6 Long-Term Care Unit, “Maria SS. dello Splendore” Hospital, AUSL 04 Teramo, 64021 Giulianova, Italy; 7 UniCamillus-Saint Camillus International University of Health Sciences, 00131 Rome, Italy; 8 Unit of Rehabilitation Treatment, Early Interventions in Mental Health, Department of Life, Health and Environmental Sciences, S. Salvatore Hospital, University of L’Aquila, 67100 L’Aquila, Italy

**Keywords:** mast cells, neurogenic inflammation, substance P, histamine, allergic diseases, attention deficit hyperactivity disorder, autism, epilepsy, anxiety, depression

## Abstract

Mast cells can release different kinds of molecules as a response to different stimuli, particularly proinflammatory mediators that contribute to neuroinflammation. The enrichment of mast cells in specific areas of the nervous system and gastrointestinal tract, together with their degranulation, histamine and neuropeptide secretion, such as substance P, and mast cell–microglia interactions, may promote neuroinflammatory signaling in neurological and psychiatric disorders during childhood and adolescence. The aim of this review is to explore the mast cell-related molecular aspects of the main neuropsychiatric disorders of the developmental period, such as ADHD, autism spectrum disorder, and epilepsy, as well as anxiety and depression. The translational analysis of molecular pathways and the relationships involved may contribute to the development of innovative and targeted therapeutic approaches.

## 1. Introduction

Mast cells have traditionally been placed at the center of allergic disease, yet their biology aligns closely with neuroimmune communication [1,2]. They are enriched in organs characterized by barrier functions, such as the skin, respiratory mucosa, gastrointestinal tract, and neurovascular interfaces, where they localize near blood vessels, lymphatics, and peripheral nerve endings. Upon activation, they can release preformed mediators within seconds and generate secondary waves of lipid mediators and cytokines, thereby producing rapid vascular and sensory effects [3,4].

In the central nervous system, mast cells display a distinctive and highly conserved distribution in both parenchyma and connective tissue coverings such as the meninge [5], being predominantly located in perivascular regions of the thalamus, hypothalamus, choroid plexus, and circumventricular organs [6,7,8]. Notably, most thalamic mast cells are located within the blood–brain barrier and in close association with blood vessels, where they can interact with neurons, glial cells, and endothelial cells, supporting their potential role in neuroimmune communication and tissue-driven inflammation [9,10,11].

Inflammatory signaling in the nervous system is not inherently pathological. Microglia and astrocytes perform homeostatic roles in synapse pruning, debris clearance, and metabolic coupling. Physiological neuroimmune signaling plays an essential role in brain development and in maintaining neural homeostasis and contributes to processes such as neuronal differentiation and circuit maturation during specific developmental stages [12]. However, when inflammatory tone becomes persistent, spatially dysregulated, or developmentally mistimed, neuronal excitability and network-level dynamics may be altered.

Interestingly, the functional consequences of immune activation depend strongly on timing, intensity, and developmental context. When excessive or temporally inappropriate during sensitive developmental windows, inflammatory signaling may contribute to long-term alterations in neuronal excitability and brain network organization. Accordingly, neuroinflammatory responses may be adaptive under physiological conditions but become maladaptive when dysregulated [13,14].

This perspective is particularly helpful for interpreting mast cells more appropriately as context-dependent modulators of neuroimmune communication rather than uniformly pathogenic drivers, particularly in the presence of stress conditions, barrier dysfunction, and multisystem comorbidities such as allergic diseases [15,16,17,18]. Neurodevelopmental disorders are defined clinically by alterations in cognition, behavior, and social functioning that emerge during childhood or adolescence, and that can persist in adulthood with variable trajectories.

Although genetic architecture provides a substantial component of risk, environmental factors, ranging from prenatal exposures to early-life infections and psychosocial stress, shape the timing and expression of symptoms. In recent years, immune–nervous system communication has become increasingly relevant to this field because inflammatory mediators can influence synaptic refinement, circuit maturation, and neurovascular function during sensitive developmental windows [19].

Two mediator systems make mast cells especially relevant to neurodevelopmental research. First, histamine is a canonical mast cell product driving itch, wheals, and vascular permeability, but it is also a brain neuromodulator involved in arousal and attentional state. Second, substance P is a neuropeptide, released during nociceptive activation and stress, capable of activating mast cells through both neurokinin receptors and the mas-related G protein-coupled receptor MRGPRX2. Together, these mediators support feed-forward neuroimmune loops that can amplify barrier inflammation, sensory hypersensitivity, and, potentially, neurovascular signaling [20].

The aim of this review is to provide a biomolecules-style synthesis that is mechanistically grounded while remaining translationally oriented. We first summarize mast cell phenotypes and activation routes relevant to neuroimmune signaling, then focus on histamine and substance P pathways and their integration with the nervous system. We subsequently discuss the relevance of these mechanisms to neurodevelopmental disorders, particularly attention deficit hyperactivity disorder (ADHD), autism spectrum disorder (ASD), epilepsy, anxiety, and depression, in which mast cell-driven immune activation and/or allergic comorbidity have been reported. Finally, we highlight open questions, methodological limitations, and priorities for biomarker development and therapeutic innovation.

The literature discussed in this review was selected through a narrative approach aimed at integrating recent and foundational studies on mast cells and neuroinflammation in neuropsychiatric disorders of the developmental period. Searches were performed in major biomedical databases, including PubMed/MEDLINE and Scopus, focusing mainly on articles published in the last two decades. Key search terms included combinations of “mast cells”, “neuroinflammation”, “histamine”, “substance P”, “allergy”, “allergic diseases”, “ADHD”, “autism spectrum disorder”, “epilepsy”, “anxiety”, and “depression”. Both human and experimental studies were considered when relevant to molecular mechanisms and neuroimmune interactions during development.

## 2. Mast Cells as Neuroimmune Sentinels

Mast cells derive from hematopoietic progenitors and complete their maturation in peripheral tissues under the influence of stem cell factor and other microenvironmental signals [21]. This developmental strategy yields organ-adapted phenotypes that differ in protease content, receptor expression, and responsiveness to local triggers. Human mast cells are often categorized into mucosal mast cells, typically enriched in respiratory and gastrointestinal mucosa and characterized by predominant tryptase content, and connective-tissue mast cells, abundant in skin and perivascular regions and containing both tryptase and chymase [22]. These distinctions are not merely taxonomic: connective-tissue mast cells display receptor programs that make them especially responsive to neuropeptides and pseudoallergic ligands, particularly for MRGPRX2 representation [23].

The classical pathway of mast cell activation involves IgE-dependent cross-linking of FcεRI by allergen, resulting in calcium mobilization, rapid degranulation, and synthesis of lipid mediators and cytokines. This mechanism is essential to understand immediate hypersensitivity, yet it does not capture the full range of mast cell behaviors in chronic inflammatory and neuroimmune conditions. Non-IgE activation pathways include complement receptors, pattern-recognition receptor signals, cytokine priming, and mechanical stress [24].

MRGPRX2 has emerged as a key non-IgE mast cell receptor with translational relevance. It is a G protein-coupled receptor activated by a broad set of cationic ligands, including host defense peptides, neuropeptides, and several drugs. Importantly, MRGPRX2 signaling does not require prior sensitization, enabling rapid degranulation patterns that align with pseudoallergic reactions. In neuroimmune terms, MRGPRX2 functions as a molecular gateway through which neural activity and stress-related mediators can access mast cell effector programs [25].

Once activated, mast cells release a repertoire of mediators with direct effects on the vascular and nervous systems. Preformed granule contents include histamine, heparin, proteases (tryptase, chymase), and stored cytokines. De novo synthesis generates leukotrienes, prostaglandins, platelet-activating factor, and multiple cytokines and chemokines. These mediators regulate leukocyte recruitment, vascular permeability, tissue remodeling, and sensory excitability. In this context, neuropeptide-driven activation may interact with the nervous system through neurons and microglia, facilitating molecular and cellular phenomena related to neuroinflammation [26].

In addition to histamine and the potential release of substance P, mast cells produce both serotonin and corticotropin-releasing hormone (CRH), supporting their role as bidirectional neuroimmune regulators, capable of both amplifying and suppressing immune responses through diverse cytokine production, such as IL-1, IL-4, IL-5, IL-10, IL-13, TNF-α, TGF-β, and IFN-γ [27,28]. In particular, mast cell-derived IL-6 and TGF-β may promote the maturation of Th17 cells and favor a proinflammatory environment and antibody production, suggesting a potential role for mast cells in autoimmune processes [29,30]. Moreover, the presence of autoantibodies against brain epitopes in mothers of children with autism spectrum disorder (ASD), as well as in many affected children, has been associated with allergic manifestations and may reflect aberrant immune responses and disruption of the blood–brain barrier (BBB), further supporting the possible involvement of mast cells in neuroimmune dysregulation and autoimmunity in ASD [29].

Dysregulation of maternal immune responses during pregnancy, including allergic or inflammatory activation, asthma, and autoimmune diseases, has been proposed as a factor influencing fetal neurodevelopment and increasing susceptibility to neurodevelopmental disorders [30,31]. In fact, mast cells are also involved in early maternal–fetal immune interactions and in critical processes such as implantation, placental vascular remodeling, trophoblast survival, and fetal growth [32,33].

Indeed, mast cells also exhibit regulatory functions: the production of IL-10 exemplifies this duality, as mast cell-derived IL-10 may exert both protective and detrimental effects on immune homeostasis depending on the immunological context [34].

## 3. Histamine at the Crossroads of Allergy and Neurodevelopment

Histamine is a biogenic amine synthesized from histidine and stored in mast cell granules at a high concentration. In peripheral tissues, histamine is synonymous with acute allergic symptom generation: it increases microvascular permeability, promotes vasodilation, and triggers itch through activation of sensory afferents and, as a neurotransmitter, is strictly associated with mast cells [35]. Clinically, these actions underpin the rapid onset of wheals, angioedema, and pruritus in hypersensitivity reactions.

From a neurobiological perspective, histamine is also a central neurotransmitter produced by a discrete population of histaminergic neurons located in the tuberomammillary nucleus of the hypothalamus. This system projects widely across the brain and modulates arousal, wakefulness, and attention [36]. Consequently, histamine receptor biology has been investigated in relation to cognitive performance and attentional control.

Four histamine receptors (H1–H4) mediate diverse outcomes with cell-type specificity. H1 receptors are strongly linked to pruritus and endothelial permeability and can also influence neuronal excitability. H2 receptors signal largely through cyclic AMP pathways and can modulate immune function and epithelial responses. H3 receptors act primarily as presynaptic autoreceptors in the nervous system and shape the release of multiple neurotransmitters, linking histamine signaling to attentional tone and behavioral state. H4 receptors are enriched on immune cells and regulate chemotaxis and inflammatory balance [37].

A key conceptual distinction is the special distribution. Neuronal histamine is released synaptically in a circuit-specific pattern, whereas mast cell histamine release occurs as an interstitial burst near vessels and nerve endings. This spatial distribution makes mast cell-derived histamine particularly effective at altering barrier permeability and sensitizing peripheral nerves. In addition, histamine can influence neurovascular units by modulating endothelial junctions and by interacting with inflammatory cytokines that prime glial cells [38]. Such effects support a model in which mast cell histamine shapes microenvironments relevant to neuroimmune communication, rather than acting solely as a peripheral symptom mediator.

Several authors have related histamine with both neurodevelopmental and neurodegenerative disorders [36]. Today, researchers mainly consider neuronal sources of histamine more than mast cell-produced histamine, whose role deserves further analysis [39].

During neurodevelopment, histamine signaling is clinically relevant. In particular, allergic comorbidity is common in pediatric populations with neurodevelopmental disorder diagnoses, and antihistamine exposure is frequent. Therefore, histamine has to be considered as a disease mechanism and as a proxy for allergic burden and medication effects that directly modulate neurobehavioral outcomes.

## 4. Substance P: Mast Cell Activation and Neurogenic Inflammation

Substance P is an 11-amino acid tachykinin peptide encoded by the TAC1 gene and enriched in capsaicin-sensitive nociceptive afferents [40]. It is released during chemical, mechanical, and thermal stimulation and serves as a key mediator of pain transmission and neurogenic inflammation. However, substance P is not restricted to neurons: immune cells and structural cells, including mast cells, lymphocytes, monocytes, macrophages, dendritic cells, endothelial cells, and epithelial cells, can also produce and release substance P. This wide distribution supports its role as a neuroimmune coordinator capable of sustaining local inflammation even when neural firing is intermittent [41].

Substance P acts locally because it is rapidly degraded by peptidases such as neprilysin. Its biological effects, therefore, depend on the proximity between sources and receptor-expressing target cells. The best-characterized receptor is the neurokinin-1 receptor (NK1R), which exists in full-length and truncated isoforms with distinct signaling properties. NK1R engagement activates phospholipase C, calcium mobilization, MAPK pathways, and transcriptional programs such as NF-κB activation, promoting chemokine and cytokine expression [42].

In addition to NK1R signaling, substance P can activate mast cells through MRGPRX2, a pathway with strong translational relevance. MRGPRX2-mediated activation produces rapid mast cell degranulation without prior sensitization and helps explain pseudoallergic reactions and symptom flares that do not align with IgE markers [43]. Once mast cells degranulate, they release histamine and proteases such as tryptase. Tryptase can activate protease-activated receptor-2 (PAR-2) on sensory neurons, increasing excitability and promoting additional neuropeptide release [44]. Thus, substance P and mast cells form a feed-forward loop in which neuropeptide release triggers mast cell activation and mast cell mediators reinforce sensory activation.

Stress pathways intersect strongly with substance P biology. Both psychological stress and systemic illness can increase substance P release and alter autonomic tone, which may lower activation thresholds for sensory nerves and mast cells [45]. This provides a mechanistic framework for stress-associated flares observed in inflammatory skin disease, asthma, gastrointestinal disorders, and pain syndromes [46,47,48,49,50,51]. In pediatric populations, where stress exposure can be high and autonomic regulation is still maturing, neuropeptide-driven immune amplification may represent an underappreciated modifier of symptom variability [52].

In the next sections, we present the main entities connected in the literature to mast cells, considering the role of produced histamine and substance P, focusing on neuropsychiatric disorders that can manifest in the neurodevelopmental period.

## 5. Attention Deficit Hyperactivity Disorder

Attention Deficit Hyperactivity Disorder (ADHD) is a neurodevelopmental disorder with onset before the age of 12, characterized by two main primary symptom domains: inattention and hyperactivity/impulsivity, inappropriate for the individual’s developmental level [53]. Numerous risk factors for ADHD have been identified and are currently under investigation: heritability, prematurity, neuroinflammation, severe brain injuries, and exposure to environmental toxins [54]. Although pathophysiology is not yet fully understood, ADHD is believed to have a multifactorial etiology, involving both genetic and environmental factors. This disorder exhibits high clinical heterogeneity, usually emerging during school age and potentially persisting across the lifespan [55]. ADHD affects 7.6% of children between 3 and 12 years of age and 5.6% of adolescents between 12 and 18 years old [56] and can remain clinically relevant in up to 2.6% of adults [57]. When diagnostic procedures are applied correctly, no significant differences in the global prevalence rates are observed [58].

As already said, the manifestations of ADHD vary considerably among individuals, and the consequences of symptoms, therefore, differ widely. In fact, inattention and hyperactivity/impulsivity are associated with relevant impairment in academic, social, and emotional functioning. Moreover, children with ADHD often share a higher risk of co-morbidity, especially other neurodevelopmental disorders, such as learning disability, intellectual development disturbances, motor and language disorders, and other mental health problems [59]. An accurate diagnosis of ADHD requires a comprehensive clinical history, in relation to established diagnostic criteria, and is sustained by clinical interviews administered to family members and teachers or work colleagues [60]. As symptoms may vary across developmental stages, management strategies differ by age: behavioral approaches in early childhood, pharmacologic and behavioral approaches in middle childhood, and pharmacologic interventions in adolescence [59]. The assessment of the effectiveness and safety of both pharmacological and non-pharmacological treatments in adulthood remains an open field for research [61].

### Mast Cells and ADHD

As previously discussed, neurobiological markers for ADHD have yet to be identified, and the mechanisms that lead to this disturbance remain partially elucidated. However, several researchers associated ADHD with inflammatory processes [62], and a possible link with allergic diseases has also been hypothesized [63]. Mast cells are well recognized as first responders to inflammatory mediators and can trigger immune responses, including in the brain [64]. Therefore, they can probably play a role in the pathogenesis of ADHD. Also, mast cell-mediated neuroflogosis has been implicated in ADHD, and interactions between mast cells and glia have been shown to exacerbate inflammation in people with ADHD [65].

This relationship has been analyzed from multiple perspectives. For instance, Y. Song et al. investigated An Shen Ding Zhi Ling, a traditional Chinese decoction used to treat ADHD symptoms, and revealed its potential to reduce hyperactivity and impulsivity in a specific rat model, possibly inhibiting mast cell activation [66]. M. R. Breach et al. examined a maternal model of allergen-induced inflammation in rats and found that the fetal brain of the offspring exhibited upregulation of microglia and an increased number of mast cells at birth, followed by abnormal social behavior and hyperlocomotion [67]. Notably, both microglia and mast cells are sensitive to sexual steroid hormones [68], suggesting that sex-dependent differences in the response to maternal inflammation could contribute to neurodevelopmental disorders [69]. Mast cells are both a primary target for estradiol and mediators of brain sex differentiation, providing a non-neuronal source for brain sexual differentiation, fundamental for specific motivated behaviors [68]. Overall, animal models associate allergic inflammation during pregnancy with mast cell levels and sex-dependent modifications in the offspring’s microglia, which, in turn, are associated with hyperlocomotion [69]. In line with this, A. A. Larson et al. reported that degranulation of mast cells in the meninges is positively associated with motor activity [69].

Another line of research examining the relation between mast cells and ADHD, originated in 2015 with the work of L.P. Heilbrun, hypothesized that chemically intolerant women had a higher likelihood of having children with ADHD or autism [70]. Building on this idea, R. F. Palmer et al. proposed that exposure to toxicants could result in alteration of mast cells, which also contribute to protection from toxicants [71], leading to induced loss of tolerance [72]. According to this theory, toxicants may induce epigenetic modifications in genes essential for mast cells’ development and activity, triggering chronic inflammation, including within the brain, thereby increasing the risk of ADHD. Palmer et al. provide evidence suggesting that chemical intolerance could be a mast cell and epigenetic-mediated risk factor for ADHD.

Following another line of research, Dian-Jeng et al. reported a possible association between maternal allergies or autoimmune diseases and an increased risk of ADHD in offspring. Although the underlying mechanisms remain unclear, genetic factors, dopaminergic dysfunction, and the transplacental passage of maternal cells have been proposed as potential contributors [73].

Recently, a group of researchers observed that, in a group of patients over 16 years of age attending a gender-affirming primary care clinic, 2.7% of the sample met diagnostic criteria for Ehlers–Danlos syndrome, compared with an estimated prevalence of 0.02% in the general population [74]. Further analysis revealed that 16.9% of this group also had a mast cell activation disorder and that 47.5% met criteria for ADHD [74]. Evidence supporting the association between Ehlers–Danlos syndrome and mast cell activation disorder is well established in the literature [75]. Emerging data suggest a similar association between Ehlers–Danlos syndrome and ADHD [76]. The mechanisms linking these two conditions remain unclear, but chronic pain has been proposed as a potential contributor to attention difficulties [76].

There is another condition that involves mast cells, common yet frequently underdiagnosed, linked to ADHD and other neuropsychiatric disorders: mast cell activation syndrome [77]. This syndrome consists of a dysregulated immune response, often associated with a mutation in genes regulating mast cell activity [78]. It results in the release of chemical mediators that produce inflammatory and allergic manifestations [79]. As key effectors of innate immunity, mast cells normally respond to injury by releasing proinflammatory cytokines, which, in turn, stimulate microglia and other cells to produce cytokines, chemokines, reactive oxygen species, and glutamate [80]. The association between mast cell activation syndrome and neuropsychiatric disorders is based on the hypothesis that dysfunction of abnormal mast cells and their mediators induces inflammation in the nervous system, both central and peripheral [81]. Patients with this syndrome exhibit higher rates of neuropsychiatric disorders compared with general age-matched controls [77]. Probably, inappropriately released mediators, such as histamine, may bind to neurons and other central nervous system cells, disrupting neuronal function [77]. The specific mediator involved could determine which neuronal populations are affected and whether neurological or psychiatric issues predominate [77].

Among the various mediators implicated, substance P deserves particular attention. In an animal model of ADHD, NK1R knockout mice, lacking the receptor for substance P, displayed locomotor hyperactivity, a phenotype also observed in wild-type mice treated with an NK1R antagonist [82]. This suggests that mutations in the NK1R gene, corresponding to the human gene TACR1, may contribute to ADHD through alterations in substance P signaling. Additional mouse studies have shown sex-specific differences, with hyperactivity in NK1R knock-out mice observed predominantly in males [83], consistent with the higher prevalence of hyperactive symptoms in boys with ADHD compared to girls [84]. Interestingly, these findings may have implications for ADHD pharmacotherapy: some studies report that methylphenidate is more effective in wild-type mice than in NK1R knock-out mice, in which it may even exacerbate symptoms [85]. Translational research in humans supported these animal findings, indicating that variations in the TACR1 gene may increase susceptibility to ADHD [86].

## 6. Autism Spectrum Disorder

Autism spectrum disorder is also a neurodevelopmental disorder. Its conceptual origins date back to 1911, when the German psychiatrist Bleuler used the term autism to describe an inwardly directed symbolic life, not readily accessible to external observers [87,88]. According to the text revision of DSM V of the APA, autism involves two core domains: deficits in social communication and the presence of restrictive and repetitive behaviors [53]. Autism spectrum disorder, or ASD, is a neurobiologically based disorder with high heritability, associated with multiple etiological factors— environmental, genetic, and immunological—that contribute to alterations in neuronal architecture, synaptogenesis, and connectivity [89]. Recent epidemiological estimates across 37 countries indicate a prevalence of approximately 97% [90]. Symptoms typically persist into adulthood, when individuals have a higher risk of developing comorbid anxiety or depression [91]. Autism has a very high heritability due to both rare variants with large effect size and common gene variants with smaller effects [92]. Clinically, ASD is characterized by difficulties in reciprocal social interaction and communication, along with restrictive and repetitive behaviors and interests, which may lead to impairment and disability, particularly when not recognized or adequately supported [93]. Diagnosis is based primarily on behavioral assessment, although several potential biomarkers, including inflammatory ones and chemokines, are currently under investigation for their potential role in autism therapy [94]. These promising new therapeutic approaches could, hopefully, be integrated one day into traditional intervention, such as cognitive behavioral therapy, emotional support, school-based services for children and adolescents, and family-centered support interventions [94].

### Mast Cells and Autism

In recent years, autism has increasingly been linked to neuroinflammation, encouraging the search for novel therapeutic interventions [95]. Particular attention has been directed toward mast cells, given their role not only in inflammatory processes but also in immunity and allergic diseases [28]. Current hypotheses propose that mast cell mediators, particularly histamine, serotonin, and cytokines, may influence inflammatory pathways that are altered in individuals with autism spectrum disorder. Interaction with immune cells and neurons may further contribute to autistic symptoms [28]. In fact, approximately 10% of individuals with ASD present comorbid atopic dermatitis, with a further increase in autism prevalence with the severity of dermatitis. This association may reflect the concurrence of neuro- and epidermal toxic factors, a hypothesis supported by the common embryonic origin of neuronal and epidermal [96]. Ehlers–Danlos syndrome, which is frequently comorbid with autism, has also been associated with mast cell activation syndrome [97]. Interestingly, evidence suggests that stimulation of mast cells driven by neuroinflammation may be particularly impactful in perinatal life, potentially impairing synaptic pruning, lowering the threshold for fear responses, and altering emotional expression, all features observed in autism [98]. Numerous studies have shown that infections during pregnancy can trigger maternal immune activation, which involves many components of innate and adaptive immunity, including mast cells, thereby increasing the risk of central nervous system disorders in the offspring [99]. We already presented evidence of a possible association between maternal immune activation and cognitive flexibility deficits, which are characteristic of different disorders, like autism spectrum [67,100,101]. Thus, immunitary, infectious, and allergic factors, even during the perinatal period, may contribute to the risk of developing such disorders. Similarly, evidence reported by Dian-Jeng et al. suggests that the link between maternal allergies and neurodevelopmental disorders may also extend to autism spectrum disorder [73].

An alteration of mast cells may also result from exposure to toxicants, mediated by epigenetic mechanisms, which alter mast cell characteristics and promote brain inflammation, thereby increasing the risk of autism [72]. An additional relevant finding comes from the work of G. A. Mostafa et al., who identified a positive correlation between nerve growth factor (NGF) and serotonin levels, released by mast cells, in a group of autistic patients [102]. The study revealed that the autistic children had NGF levels above the normal range, potentially contributing to neurogenic inflammation [102,103].

**Figure 1:**
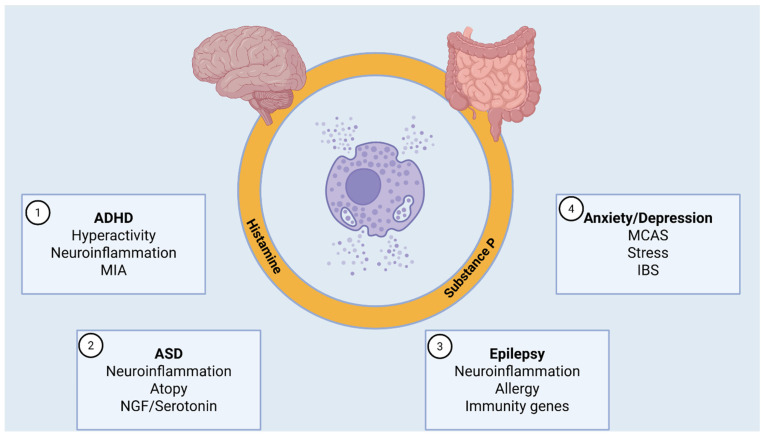
Mast cell-related neuroinflammatory mechanisms and barrier dysfunction in neuropsychiatric disorders in childhood and adolescence. Schematic representation of the potential role of mast cells and their principal mediators, including histamine and substance P, in neuroinflammatory mechanisms associated with barrier dysfunction in selected neuropsychiatric disorders of the developmental period. The stylized illustrations of the brain and the gastrointestinal tract provide an anatomical context for alterations of the blood–brain barrier and intestinal barrier, which may facilitate immune–neural interactions and contribute to inflammatory signaling relevant to neuropsychiatric manifestations. The figure summarizes disorder-specific features potentially associated with mast cell activation: (1) Attention Deficit Hyperactivity Disorder (ADHD), characterized by hyperactivity, neuroinflammation, and a possible contribution of maternal immune activation (MIA); (2) Autism Spectrum Disorder (ASD), associated with neuroinflammation, atopy, and altered levels of nerve growth factor (NGF) and serotonin; (3) Epilepsy, in which neuroinflammatory processes, allergic mechanisms, and immunity-related genes may contribute to disease susceptibility; (4) Anxiety and depressive disorders, frequently associated with mast cell activation syndrome (MCAS), stress, and irritable bowel syndrome (IBS). ADHD: Attention Deficit Hyperactivity Disorder, ASD: Autism Spectrum Disorder, MIA: Maternal Immune Activation, NGF: Nerve Growth Factor, MCAS: Mast Cell Activation Syndrome, IBS: Irritable Bowel Syndrome. Created in BioRender. Ginaldi, L. (2026) https://BioRender.com/60bbzpq (accessed on 31 January 2026).

A deeper understanding of the relationship between autism and mast cell activation may be crucial for elucidating the pathogenesis of this disorder and identifying novel molecular targets for intervention [81,104]. Significant evidence also highlights the role of substance P (Figure 1) and other tachykinins in ASD. For example, researchers have shown that tachykinin 1-expressing neurons in the periaqueductal gray send excitatory projections to oxytocin-producing ones in the paraventricular hypothalamus, thereby promoting social behaviors, which are impaired in autism spectrum disorder [105]. In fact, neuropeptides are essential for brain development, and their dysregulation could contribute to neurotoxic effects [106]. Further supporting this connection, Mostafa et al. identified a positive linear relationship among serum mercury levels (known for being neurotoxic), neurokinin A, and scores on the Childhood Autism Rating Scale. These findings suggest that high mercury levels may induce neuroinflammation with increased levels of tachykinin A, potentially contributing to ASD pathogenesis [107].

## 7. Epilepsy

Epilepsy is a neurological disease defined by the occurrence of two unprovoked epileptic seizures at least 24 h apart or by a single unprovoked seizure accompanied by a recurrence risk of at least 60% or by the diagnosis of an epilepsy syndrome [108]. It is characterized by synchronous activation of neuronal populations, resulting in a surge of excessive electrical activity that may produce involuntary movements, sensations, emotions, and/or behavior [109]. It has a prevalence of around 1% of the global population [110] and significantly affects children, with substantial repercussions on quality of life [111]. A large body of studies indicates that epilepsy has a strong genetic component, involving also epigenetic mechanisms, underlying susceptibility and pathogenesis for this disease [112]. Nevertheless, the etiology of epilepsy also encompasses structural, metabolic, infectious, and autoimmune factors [113]. Clinical history and physical examination form the foundation of diagnosis, but specialized investigations and electrophysiological exams are essential to exclude epilepsy mimics [113]. Therapy for epilepsy includes medical therapies, surgery, special devices for nervous stimulation, and dietary approaches [114]. Potentially novel therapeutic strategies involve immunomodulatory interventions, based on the hypothesis that inflammatory pathways contribute to the pathogenesis [115], as well as gene therapies, for which epilepsy is considered an ideal target [116]. Increasing attention is being directed toward precision medicine approaches to optimize treatment selection, especially for genetically determined forms of epilepsy, although further studies are needed in this direction [117].

### Mast Cells and Epilepsy

Multiple pieces of evidence suggest an involvement of mast cells in epilepsy. For example, Park G. et al. reported that Cicadidae Periostracum, an insect-derived product traditionally used in Asian medicine to treat epilepsy, was found to alleviate atopic dermatitis symptoms in a mouse model. Histopathological analyses showed that this effect was associated with a reduction of mast cells’ infiltration, immunoglobulin E, and histamine [118]. The authors suggested that these effects may result from the regulation of inflammasome activation through inhibition of nucleotide-binding oligomerization domain-like receptor protein 3 (NLRP3) [118]. In another study, Pan H. et al. found that children with allergic rhinitis had a 76% increased risk of developing epilepsy, with an additional 21% increase in boys compared to girls, and a younger age at epilepsy diagnosis [119]. They found that different studies suggest that mast cell degranulation due to allergic triggers may induce focal brain inflammation and, thereby, contribute to epileptogenesis [120]. Their findings also indicated that children with allergic rhinitis had a higher risk of developing epilepsy, ADHD, and autism [119].

The association between mast cells and epilepsy has also been explored from a genetic perspective. Epileptic subjects exhibit altered immune profiles, with an increase of activated mast cells, associated with specific key genes such as FGD3 and SSH2 [121]. In fact, many genes differentially expressed in epilepsy are related to immune responses [122]. Although the precise role of immunity-related genes in epilepsy remains unclear, some evidence points to epidermal growth factor receptor (EGFR), given the observed correlation between levels of EGFR and infiltration of immune cells, including mast cells [123]. Similarly, histamine, TNF-α, and IL-2 may contribute to seizures in children, and modulation of mast cells’ activation may exert neuroprotective effects [124]. These findings suggest that mast cells are a possible target for reducing neuroinflammation associated with epilepsy and that a probable crosstalk between cerebral histaminergic neurotransmission and histamine released by mastocytes exists [125]. In contrast to these observations, E. Kilinc et al. reported that mast cell degranulation exerted an anticonvulsant effect in an animal model, possibly mediated by 5-HT [126]. Several explanations may account for this discrepancy: heterogeneous mediator content of mastocytes, release of both proinflammatory and anti-inflammatory mediators by mast cells, regional differences in receptor expression across brain areas, and mast cell-induced stimulation of anti-inflammatory mediators, such as 5-HT [126]. Nevertheless, further research is needed to clarify the precise role of mast cells in epilepsy.

Finally, it is worth considering that epilepsy has been strongly linked to substance P. This neuropeptide is thought to contribute to seizure induction via the NK1 receptor [127]. This clarifies why neuropeptide receptors are possible targets for antiepileptic therapy [128]. Some evidence suggests that substance P expression may be induced by seizure activity in an age-dependent manner [129]. Other studies hypothesize that substance P facilitates epileptic activity in the hippocampus while, paradoxically, protecting from seizures when activating the substance P receptor on specific interneurons [130]. These studies underscore the need for further research to comprehend the complex role of substance P in the pathogenesis of epilepsy.

## 8. Anxiety and Depression

Anxiety and depression are both common conditions in the developmental period. They are characterized, respectively, by difficulties in managing worries and fears that interfere with participation in daily activities, and in disturbances of mood with core symptoms of sadness and irritability [131]. These conditions may emerge in preschool age and have been associated with both genetic and environmental factors [131]. Diagnostic assessment of anxiety and depression in children and adolescents relies on a comprehensive evaluation of medical history, identification of core symptoms, and analysis of developmental functioning. This process is supported by standardized rating scales and analysis of risk and severity factors [132,133]. Early intervention is crucial for improving outcomes and typically involves a multimodal approach consisting of psychoeducative and psychotherapeutic treatment and pharmacological interventions, especially SSRIs [134,135].

### Mast Cells and Anxiety and Depression

Several studies have explored the potential relationship between mast cells and anxiety or depression in young people.

A study examined the prevalence of depressive symptoms in 125 individuals with mast cell activation syndrome and found that depression was more common among younger participants, with symptom severity increasing proportionally to illness intrusiveness [136]. The authors attributed this association primarily to psychosocial factors, including illness burden, adverse psychological states, and limited interpersonal and intrapersonal resources [136].

In a different line of research, Houghton et al. considered immune alteration in people with food allergies as a possible contributor to broader general effects, in particular on the nervous system, including manifestations of anxiety [137]. This finding aligns with literature demonstrating a bidirectional connection between mast cells and neurons in animal models [138,139]. Several mechanisms have been proposed to explain this neuroimmune communication. Firstly, mast cells are located close to neurons in the gut, especially in allergic models; in fact, the microbiota–gut–brain axis exerts direct effects on brain function through neural, metabolic, and immune mechanisms [140], with dysbiosis consistently associated with neuropathological disorders [141]. Secondly, vagus nerve stimulation reduces mast cell number and degranulation, whereas vagotomy can prevent anaphylaxis in IgE-sensitized animals. Thirdly, mast cells’ tryptase, in animals, stimulates neuronal release of calcitonin gene-related peptide (CGRP) and substance P via protease-activated receptor-2. Fourthly, exposure to supernatant from activated mast cells activates neurons in human ex vivo models Lastly, mast cell depletion prevents allergen avoidance behavior in sensitized animals [137].

Notably, it is ascertained that children, adolescents, and young adults with food allergies exhibit higher rates and more severe forms of anxiety [142,143,144,145]. However, further research is needed to clarify the specific neuroimmune pathways underlying the relationship between food allergies and anxiety [137].

Finally, the link between mast cells and anxiety or depression in young people has also been investigated in the context of gastrointestinal disorders. In a sample of youths aged 8–17 years with irritable bowel syndrome (IBS), Singh et al. reported an increased number of mast cells in the descending colon and rectosigmoid, associated with anxiety and depression [146]. Similarly, Friesen et al. found that nausea in children and adolescents with functional gastrointestinal disorders was associated with increased mast cell density and higher levels of self-reported anxiety and depression [147]. Nonetheless, it is worth considering that these associations do not necessarily imply causation, and further studies are required to elucidate characteristics and mechanisms underlying these relationships (Table 1).

## 9. Discussion

Mast cells are present in meningeal and perivascular compartments and can interact with endothelial cells, neurons, astrocytes, and microglia. These interactions are relevant for neurodevelopment because the BBB and the neurovascular unit regulate exposure of the developing brain to peripheral mediators. Mast cell mediators can influence BBB behavior through multiple mechanisms. Histamine is vasoactive and can alter endothelial junctional dynamics. Proteases and cytokines can modify the extracellular matrix and induce adhesion molecule expression, promoting immune cell trafficking. Substance P itself can modulate endothelial activation and contribute to plasma leakage. When combined, these pathways may facilitate increased peripheral-to-central signaling under conditions of chronic barrier inflammation.

Microglia, the resident immune cells of the CNS, respond to cytokine environments and can adopt primed states characterized by increased basal inflammatory signaling and altered synaptic pruning behavior. Mast cell-derived cytokines can contribute to microglial activation, while microglial mediators can, in turn, shape neuronal excitability and synaptic remodeling.

Moreover, mast cells at neurovascular interfaces can translate peripheral immune activity into local endothelial and glial changes, potentially modifying the threshold for neuroinflammation. This model aligns with emerging views that neurodevelopmental disorders are not solely neuronal circuit disorders but can involve multi-system interactions encompassing immunity, barrier function, and neuroendocrine regulation [10].

Translating mast cell–histamine–substance P biology into pediatric care requires biomarkers that capture pathway activity with sufficient temporal resolution. Traditional allergy measures, such as total IgE and allergen sensitization tests, capture only a subset of activated mast cells and do not reflect neuropeptide-driven pathways. Similarly, single-time-point serum tryptase measurements may miss episodic mediator release or may not reflect protease-dominant activation patterns.

A promising approach could be the one that combines symptom-based phenotyping with mediator panels. Candidate measures include histamine metabolites, tryptase isoforms or protease activity markers, and substance P levels, ideally collected during symptom flares. Because substance P is rapidly degraded, sampling timing and pre-analytical handling are critical. Tissue-based assessments, such as MRGPRX2 expression in skin or mucosal biopsies, could provide additional mechanistic resolution in selected clinical scenarios.

Therapeutic development must account for the neuroinflammatory loops we have shown. NK1R could be modulated through antagonists with potential preclinical anti-inflammatory effects [148]. However, in clinical contexts, the parallel activation routes, including MRGPRX2, may maintain mast cell activation. Targeting MRGPRX2 is conceptually attractive and, in preclinical trials, novel small-molecule antagonists may inhibit mast cell degranulation [149]; however, to date, there are no approved drugs specifically designed to modulate these pathways.

Histamine receptor modulation remains clinically accessible, yet sedating antihistamines can complicate neurobehavioral outcomes, highlighting the need for receptor-selective and age-appropriate strategies.

Under a translational point of view, highlighting the mechanisms through which mast cells are linked to neuropsychiatric disorders of the developmental period potentially represents a basis to individuate novel therapeutic strategies.

Among non-pharmacologic approaches, improving the barrier integrity of the skin and the gastrointestinal tract, and reducing inhalant, food, and contact allergen exposure can reduce neurogenic amplification and improve daytime function. In pediatrics, such approaches may yield benefits even when mechanistic biomarkers are incomplete. Ultimately, the goal is to identify modifiable neuroimmune circuits that contribute to symptom burden in selected children and adolescents with neuropsychiatric disorders.

On the other hand, regarding pharmacological approaches, different paths have been explored. Epilepsy represents a prototype to enlighten us on how anti-inflammatory interventions could play a pivotal role in the therapy of neuropsychiatric disorders of children and adolescents [126]. In fact, in the analysis of Ramos et al., five anti-inflammatory drug categories were revealed to be promising in treating the disturbance. First of all, histaminergic ligands could reduce the duration and severity of seizures, preventing mast cell degranulation and histamine release. Second, interleukin receptor antagonists could be effective in terminating status epilepticus in patients with refractory forms. Third, non-steroidal anti-inflammatory drugs (NSAIDs) could reduce neuronal injury through COX-2 modulation and enhance the effects of antiseizure medications. Fourth, inhibitors of microglial activators, for example, gabapentin, could reduce microglial activation and, consequently, increase convulsive threshold. Last, some herbal medications, as well as cannabidiol, are considered to have antioxidant and anti-inflammatory effects with a potential effect on seizure control.

It is notable that many perspectives, both non-pharmacological and pharmacological, are now showing in the landscape of promising interventions. Therefore, further studies are necessary to comprehend the real role of anti-inflammatory-based strategies and to individuate new pathways to ensure better control of specific pathological conditions.

## 10. Conclusions and Future Perspectives

Mast cells occupy a strategic position at the intersection of immunity and the nervous system. Through rapid mediator release and responsiveness to neuropeptides, they can shape vascular permeability, barrier integrity, and sensory excitability. Histamine and substance P represent two highly connected mediator axes that link mast cell biology to neurogenic inflammation, and potentially to neurovascular and glial signaling. In childhood and adolescence, neuropsychiatric disorders, where immune dysregulation and allergic comorbidity are common and symptom trajectories are highly variable, these pathways may function as modulators that amplify itch, pain, sleep disruption, and stress sensitivity.

Current evidence does not support mast cells as singular causal drivers of ADHD, ASD, epilepsy, anxiety, depression, or other neurodevelopmental conditions. Nonetheless, the mechanistic context reviewed here provides a coherent explanation for clinical clustering between allergic disease and neurobehavioral symptoms, and highlights actionable translational targets. Future progress will depend on biomarker-resolved endotyping and experimental models that reproduce neurovascular niches and nerve–mast cell proximity. A precision neuroimmune approach, aimed at reducing maladaptive amplification rather than suppressing immunity broadly, may ultimately improve quality of life and functional outcomes in selected pediatric subgroups.

## Figures and Tables

**Table 1:** Mast cell/histamine-related evidence across neuropsychiatric disorders in the developmental period. ADHD, attention-deficit hyperactivity disorder; ASD, autism spectrum disorder; BBB, blood–brain barrier; GI, gastrointestinal; MCAS, mast cell activation syndrome; MIA, maternal immune activation; NGF, nerve growth factor; 5-HT, serotonin.

Disorder	Key Findings (Human Studies/Models)	Proposed MC/Substance P/Histamine Role	Refs.
ADHD	Maternal immune activation (MIA) is implicated in neurodevelopmental vulnerability; chemical intolerance in parents predicts risk for ADHD in offspring; knock-out mice for NK1R expressed hyperactivity	Neuroimmune priming; mediator-driven inflammation as a contributor to symptom domains; impairment in substance P pathway	[70,72,82,101]
ASD	Mast cell–cytokine axis and perinatal mast cell activation/neuroinflammation are discussed in ASD frameworks; chemical intolerance/environmental sensitivity associated with ASD/ADHD risk	Neuroimmune dysregulation; mast cell mediators (histamine/cytokines) as modulators of neuroinflammation	[70,72,97,100,104]
	Elevated NGF and serotonin-related signals reported in autistic children; NGF–serotonin correlation proposed; high neurokinin levels reported in subjects with ASD	Mediator imbalance (NGF/5-HT) potentially linked to neurogenic inflammation; neuronal signaling	[102,105,107]
Epilepsy	Atopy/allergic rhinitis associated with increased epilepsy risk; experimental and genetic data suggest immune–mast cell involvement; mixed pro-/anti-convulsive effects reported; substance P is a possible inducer of seizures	Inflammation and BBB/immune interactions; mast cell mediator effects on excitability (histamine/5-HT); activation of NK1R	[96,115,119,126,127]
Anxiety and Depression	Depressive symptoms reported in MCAS; food allergy–anxiety links and gut mast cell density associated with anxiety/depression in functional GI disorders; substance P is released in anxiety disorders in people with food allergy	Gut–brain axis involvement; stress sensitivity and mast cell–neuron crosstalk	[136,137,146,147]

## Data Availability

No new data were created or analyzed in this study. Data sharing is not applicable to this article.
